# The Role of Hemoproteins: Hemoglobin, Myoglobin and Neuroglobin in Endogenous Thiosulfate Production Processes

**DOI:** 10.3390/ijms18061315

**Published:** 2017-06-20

**Authors:** Anna Bilska-Wilkosz, Małgorzata Iciek, Magdalena Górny, Danuta Kowalczyk-Pachel

**Affiliations:** Chair of Medical Biochemistry, Jagiellonian University Collegium Medicum ,7 Kopernika Street, Kraków 31-034, Poland; miciek@cm-uj.krakow.pl (M.I.); mbgorny@cyf-kr.edu.pl (M.G.); dkowalczyk@cm-uj.krakow.pl (D.K.-P.)

**Keywords:** hemoglobin, hydrogen sulfide, myoglobin, neuroglobin, thiosulfate

## Abstract

Thiosulfate formation and biodegradation processes link aerobic and anaerobic metabolism of cysteine. In these reactions, sulfite formed from thiosulfate is oxidized to sulfate while hydrogen sulfide is transformed into thiosulfate. These processes occurring mostly in mitochondria are described as a canonical hydrogen sulfide oxidation pathway. In this review, we discuss the current state of knowledge on the interactions between hydrogen sulfide and hemoglobin, myoglobin and neuroglobin and postulate that thiosulfate is a metabolically important product of this processes. Hydrogen sulfide oxidation by ferric hemoglobin, myoglobin and neuroglobin has been defined as a non-canonical hydrogen sulfide oxidation pathway. Until recently, it appeared that the goal of thiosulfate production was to delay irreversible oxidation of hydrogen sulfide to sulfate excreted in urine; while thiosulfate itself was only an intermediate, transient metabolite on the hydrogen sulfide oxidation pathway. In the light of data presented in this paper, it seems that thiosulfate is a molecule that plays a prominent role in the human body. Thus, we hope that all these findings will encourage further studies on the role of hemoproteins in the formation of this undoubtedly fascinating molecule and on the mechanisms responsible for its biological activity in the human body.

## 1. Introduction

**Figure 1:**
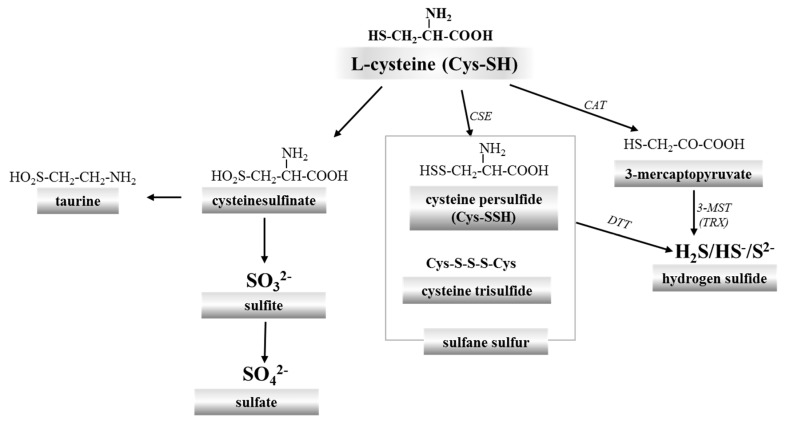
Two l-cysteine transformation pathways: the aerobic path leads to taurine and sulfate, while the anaerobic route to sulfane sulfur-containing compounds and hydrogen sulfide. CSE: cystathionine γ-lyase; CAT: cysteine aminotransferase; 3-MST: 3-mercaptopyruvate sulfurtransferase; TRX: thioredoxin; DTT: dithiothreitol.

**Figure 2:**
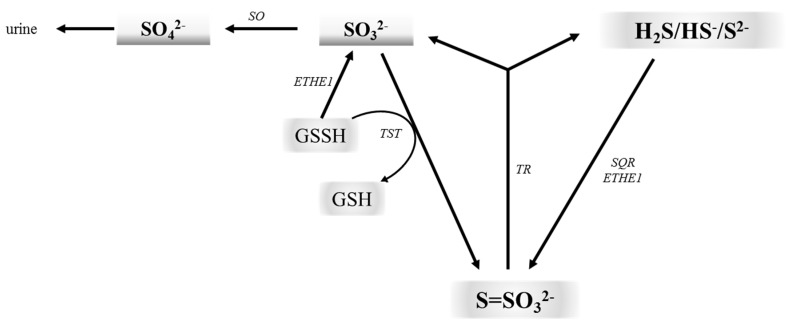
The thiosulfate cycle links aerobic and anaerobic metabolism of cysteine. H_2_S can be oxidized by SQR and ETHE1 to thiosulfate. Sulfane sulfur can be transferred from GSSH to sulfite leading to thiosulfate formation. Sulfite can also be further oxidized to sulfate by SO. On the other hand, thiosulfate may be converted back to H_2_S and sulfite by TR. SO: sulfite oxidase; ETHE1: ethylmalonic encephalopathy protein 1; TST: rhodanese; TR: thiosulfate reductase; SQR: sulfide:quinone oxidoreductase.

Cysteine is the main source of sulfur in the animal and human body. It is metabolized via two pathways. The first one, called the cysteinesulfinate-dependent (aerobic) pathway, is a series of processes leading to taurine and inorganic sulfate (SO_4_^2−^). The second path is independent of cysteinesulfinate (anaerobic) and is a source of sulfane sulfur-containing compounds and hydrogen sulfide/sulfide (H_2_S/S^2−^) (Figure 1). In general, sulfur metabolism in animals consists in its oxidation to the highest +6 oxidation state. Thus, SO_4_^2−^ can be considered the final product of sulfur metabolism in animals. The sulfane sulfur is a labile reactive sulfur atom in the 0 or –1 oxidation state, covalently bound to another sulfur atom which can easily leave the structure of the compound and can readily react with various acceptors, such as reduced glutathione (GSH) and other thiols (RSH) or cyanide (CN^−^) [1,2,3] (Figure 2). Some other compounds, such as the outer sulfur atom of thiosulfate (S=SO_3_^2−^) and elemental sulfur (S_8_), also possess properties characteristic of sulfane sulfur [4]. In the literature, aside from sulfane sulfur, the term “bound sulfur” is also used. This term describes sulfur that can be released as H_2_S by reducing agents, such as dithiothreitol (DTT) [5,6,7]. It is believed that “bound sulfur” can be a fraction of sulfane sulfur [4].

In 1996, hydrogen sulfide (H_2_S) was first evidenced to be produced endogenously in mammalian tissues, suggesting that it could have physiological functions [8]. It is synthesized from l-cysteine by cystathionine γ-lyase (EC 4.4.1.1; CSE) and cysteine aminotransferase (EC 2.6.1.3; CAT) in cooperation with 3-mercaptopyruvate sulfurtransferase (EC 2.8.1.2; 3-MST) [9]. H_2_S has a half-life in vivo of several minutes [10]. It is currently known that H_2_S is an important biological messenger molecule that transmits signals by forming persulfide bonds (SSH) in proteins or in low-molecular thiols [11,12,13]. In this context, it is interesting to look at sulfide:quinone oxidoreductase (EC 1.8.5.4; SQR) involved in mitochondrial H_2_S oxidation. This enzyme catalyzes a two-electron oxidation of H_2_S to the sulfane sulfur containing persulfide SQR-SSH. This sulfane sulfur is then transferred onto different nucleophilic acceptors, including reduced glutathione (GSH), which is transformed into glutathione persulfide (GSSH). The sulfur of GSSH may by then oxidized to sulfite by ethylmalonic encephalopathy protein 1 (EC 1.13.11.20; ETHE1) or transferred to sulfite (SO_3_^2−^) by rhodanese (EC 2.8.1.1; thiosulfate:cyanide sulfurtransferase, TST) to form thiosulfate (Figure 2). Thus, thiosulfate is formed as a result of reaction between intermediates of two metabolic routes, namely sulfite and sulfide. Sulfite is further oxidized to sulfate by sulfite oxidase (EC 1.8.3.1; SO), whereas thiosulfate may be converted back to H_2_S and sulfite by thiosulfate reductase (EC 2.8.1.3; TR) [14,15,16,17]. It appears that thiosulfate may be considered as a safe nontoxic storage form of H_2_S in the body. The unconventional experiments by Koj and Frendo [18], Koj et al. [19] and Skarżyński et al. [20] clearly demonstrated a central role of thiosulfate as a key intermediate in the H_2_S metabolism.

It seems that thiosulfate production can delay irreversible H_2_S oxidation to SO_4_^2−^ excreted in urine. These processes occurring in mitochondria are termed a canonical H_2_S oxidation pathway. However, recently new chemical interactions between H_2_S and hemoproteins have been described which yield thiosulfate and iron-bound hydropersulfides. This process is called a non-canonical H_2_S oxidation pathway [21]. Although it has been mostly examined in relation to H_2_S detoxification, the thiosulfate formation as a biologically important molecule appears to be the essence of this process. Therefore, looking at H_2_S catabolism engaging hemoproteins in this context is interesting and inspiring but it has still received little attention.

The aim of the present work was to briefly recapitulate the current state of knowledge on interrelationships between H_2_S and hemoproteins: hemoglobin (Hb), myoglobin (Mb) and neuroglobin (Ngb) and to highlight a potential biological significance of thiosulfate as a direct product of H_2_S oxidation by hemoproteins.

## 2. Red Blood Cells, Hemoglobin and Hydrogen Sulfide

Hemoglobin of adults (HbA) is a hematoporphyrin built of four polypeptide chains (two α and two β). The α chain is composed of 141 amino acids while the β chain of 146 amino acids. Each chain binds one heme molecule. The main function of hemoglobin consists in oxygen transport from the lungs to the tissues. One Hb molecule can bind four oxygen molecules. Thanks to Hb, oxygen carrying capacity of one liter of blood is 250 mL of oxygen while without Hb one liter of blood could transport only 5 mL of oxygen. Hb also participates in carbon dioxide (CO_2_) transport from tissues to the lungs, carrying ca. 60% of the total amount of CO_2_ exchanged by red blood cells (RBCs) during breathing. Moreover, Hb is the most important transport protein of nitric oxide (NO). Hb exhibits the greatest affinity for NO in relaxed (R) conformation i.e. during full oxidation.

Under physiological conditions, the reduced form called deoxyhemoglobin (deoxyHb) binds oxygen in the lungs and is transformed into oxyhemoglobin (oxyHb) which alters oxidation state of the centrally located ferrous ion (Fe^2+^). OxyHb is a stable form but under physiological conditions even 3% of oxyHb per 24 h is oxidized to methemoglobin (ferric hemoglobin, MetHb) in which centrally located iron is in the +3 oxidation state (Fe^3+^) [22]. MetHb is unable to transport oxygen and CO_2_. Hb participates also in many other physiological processes. It is not an enzyme but shows pseudoenzymatic activity of catalase, peroxidase, monooxygenase and esterase, and also *O*- and *N*-demethylase [23,24]. The presence of 3-MST in RBCs, which is an enzyme engaged in the formation and transport of sulfane sulfur and H_2_S (Figure 1) and the fact that these cells do not contain mitochondria which house the canonical H_2_S oxidation machinery, suggest that Hb can be also responsible for maintaining H_2_S and sulfane sulfur homeostasis in the circulatory system. The reaction of H_2_S with Hb to produce sulfheme derivatives is commonly known and involves the covalent addition of sulfide to one of the pyrrole rings [25]. Sulfhemoglobin (SulfHb) is a green-pigmented molecule containing a sulfur atom in one or more of the porphyrin rings. Normally, SulfHb is not present in the blood, and its levels greater than 60% can lead to death due to tissue hypoxia. Sulfhemoglobinemia is a hematological condition accompanying treatment with some drugs, including co-trimoxazole, phenacetin, phenazopyridine and metoclopramide [26]. Sulfhemoglobinemia can also result from the presence of H_2_S-producing bacteria in the intestine [27]. Recently, a case of sulfhemoglobinemia was described in a child with chronic constipation and urinary tract infection [27].

In adults, Hb concentration in the whole blood averages 2.3 mM while a mean corpuscular Hb concentration (MCHC) is ca. 5.3 mmol/L RBCs. MetHb accounts for 1−3% of the total Hb, and its concentration in the whole blood is 25–75 μM (corresponding to 100–300 μM ferric heme) and 53–159 μM in 1 liter of RBCs (corresponding to 212–636 μM ferric heme), respectively. Considering that RBCs occupy ca. 40–50% of blood volume, it is obvious that MetHb plays a significant role in H_2_S oxidation. Literature data demonstrated that ca. 3 moles of H_2_S were inactivated by one mole of MetHb [28]. However, data on the concentration of H_2_S in human plasma and tissues are equivocal. Olson underlined that the reports of 2001–2009 indicated its high concentration in the body, estimated even at 300 µM, while since 2010 a majority of studies revealed a much lower concentration [29]. At present, it is believed that the true free H_2_S concentration in human plasma and tissues is in low micromolar or even nanomolar range [9].

**Figure 3:**
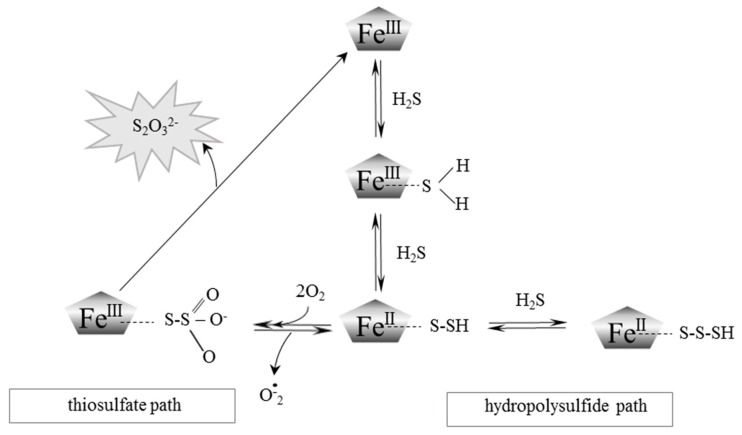
A schematic representation of the postulated mechanism for the MetHb and MetMb-dependent oxidation of H_2_S to thiosulfate and heme iron-bound persulfides. Modified from Vitvitsky et al. [21] and Bostelaar et al. [38].

H_2_S differs from other gasotransmitters (NO and CO) in its ability to dissociate. Its pKa_1_ is 6.89, and pKa_2_ is 19.0 and in physiological conditions, i.e. at pH 7.4 in aqueous solutions, 20% of hydrogen sulfide remains undissociated and 80% is dissociated to HS^−^ anion while S^2−^ anion concentration is extremely low. The rate constant for H_2_S binding to ferric Hb (Fe^III^-Hb) increases with decreasing pH [21,30,31]. This fact suggests that H_2_S rather than more abundant HS^−^ anion initially interacts with MetHb. The course of this process was described by Vitvitsky et al. [32]. As presented in Figure 3, H_2_S is oxidized by MetHb to form thiosulfate and hydropolysulfie.

## 3. Properties of Myoglobin and Its Interaction with Hydrogen Sulfide

Mb is a monomeric heme protein found in most animal muscles in a concentration of 0.5–1% by weight [33]. It contains one heme group per protein molecule. Molecular weight of human Mb is ca. 17,800 Da. Similar to Hb, Mb is a cytoplasmic protein that binds oxygen on a heme group. Although heme groups of both proteins are identical, Mb has a higher affinity for oxygen than Hb. The partial pressure of oxygen (pO_2_) in the blood, at which Hb saturation is 50% (P_50_), is about 26 mmHg for a healthy person, while Mb reaches 50% saturation already at pO_2_ = 1 mmHg. This difference is related to its different role: whereas Hb transports oxygen, Mb function is to store oxygen and release it during periods of its deprivation in muscle tissues. Similar to Hb, Mb occurs in the body in three characteristic physiological forms: oxyMb and deoxyMb, in which centrally located iron is in the +2 oxidation state (Fe^2+^) and ferric Mb (MetMb), in which centrally located iron is in the +3 oxidation state (Fe^3+^).

Mb was the first protein whose 3D structure was revealed using X-ray crystallography [34]. The amino acids sequence identity between the α-chain, β-chain of Hb and the chain of Mb is approximately 12%. Thus, it is quite unexpected that the tertiary structures of all three polypeptide chains are surprisingly similar [35,36]. It means that very different amino acid sequences can encode truly similar spatial structures determining biological function of proteins.

Therefore, it is not surprising that, similar to in the case of MetHb, an interaction between MetMb and H_2_S that leads to catalytic turnover of H_2_S has been discovered [21]. It was also demonstrated that whale sperm MetMb bound H_2_S in vitro (*K*_D_ = 18.5 μM at pH 7.5, at a temperature of 20 °C) [37], similar to human MetHb (*K*_D_ = 17 μM at pH 7.4, at a temperature of 37 °C) [38]. The transformation of H_2_S by MetMb is similar to that of MetHb and also leads to the formation of thiosulfate and hydropolysulfide [38] (Figure 3).

## 4. Interactions of Neuroglobin with Hydrogen Sulfide

Ngb is a member of the hemoprotein family which is expressed in the brain, mostly in the subthalamic nucleus, frontal lobe, thalamus and occipital pole [35]. Ngb was also identified in other tissues, such as the gastrointestinal tract, lungs, some endocrine tissues, spinal cord, lymph nodes and in the peripheral nervous system [39,40,41]. It has been demonstrated that Ngb is the third protein, besides Hb and Mb, reversibly binding oxygen via the Fe^2+^ ion of the heme group in humans and other vertebrates [39,42]. Ngb is a six-coordinate hemoprotein, with the heme iron coordinated by two histidine residues. In terms of physicochemical properties, Ngb shows a greater similarity to Mb because it is a monomer with molecular weight of 17,000 Da composed of 151 amino acids and characterized by a high affinity for oxygen (half saturation pressure, P_50_ = 1–2 mmHg at 37 °C) [42]. Hence, it can be concluded that Ngb is analogous to Mb. However, Ngb shares little amino-acid sequence similarity with Mb (<21% identity) [39]. Ngb displays also a modest sequence homology with Hb (<25% identity) [39]. The physiological role of Ngb is still elusive. Currently, it is assumed that Ngb is an endogenous neuroprotective protein, active against several brain injuries, including hypoxia, ischemia, toxicity, and oxygen/nutrient deprivation [43]. In vitro studies showed that H_2_S bound very tightly to the heme group of human ferric Ngb (MetNgb). The bimolecular rate constant for H_2_S binding to MetNgb was about 35 and 1000 fold slower than for MetHb and MetMb, respectively [30].

Ruetz et al. [44] observed that H_2_S oxidation to sulfate and thiosulfate in the brain homogenate was insensitive to stigmatellin, an inhibitor of complex III. On the other hand, it is known that the level of mRNA (and protein) encoding SQR in the brain is very low [45]. Thus, it can be expected that a non-canonical pathway involving MetNgb plays the main role in H_2_S oxidation in the brain.

## 5. Thiosulfate: From a Traditional Photographic Darkroom to Modern Medicine

Thiosulfates are salts of unstable thiosulfuric acid (H_2_S_2_O_3_), of which the best known is sodium salt, i.e. sodium thiosulfate (STS, Na_2_S_2_O_3_).

STS was widely used in traditional photography to fix black and white negatives and prints after the developing stage. This compound has long been known also to physicians and pharmacologists. Historically, it was used in the treatment of cyanide poisoning. Transformation of toxic cyanide to much less toxic thiocyanate (rhodanate) with the use of an outer sulfur atom of STS is catalyzed by TST. Thiocyanate is excreted by the kidneys. Cyanides are widespread as components of many plants and can cause poisonings following ingestion of some plant products. However, small amounts of cyanides are normally detoxified with the use of endogenous thiosulfate.

Bijarnia et al. observed a beneficial effect of STS in a chronic rat model of ethylene glycol (EG)-induced hyperoxaluria. In particular, STS significantly reduced crystal load and preserved creatinine clearance, tissue superoxide dismutase (SOD) and urine 8-isoprostaglandin levels. In vitro studies showed the ability of STS to scavenge oxalate-induced reactive oxygen species (ROS), to reduce cell-released hydrogen peroxide and to preserve SOD activity. According to those authors’ opinion, antioxidant properties of STS were responsible for efficacy of this compound in alleviating EG-induced hyperoxaluria in rats [46]. It has also been indicated that α-tocopherol and STS supplementation during antibiotic therapy in patients with infiltrative pulmonary tuberculosis allows for a considerable reduction of antibiotic load. The authors suggested also the antioxidant properties of STS as the mechanism explaining this finding [47]. Thomas and McGinnis [48] reported the safe co-administration of intrathecal sodium nitroprusside and STS in 10 patients with secured ruptured cerebral aneurysms in the intensive care unit, without the use of cerebral angiography for vasospasm treatment.

In the last decade, we have witnessed an increasing interest in STS as a drug in patients suffering from calciphylaxis. Calciphylaxis was first described by Selye et al. [49]. This disease is characterized by the accumulation of calcium deposits in small and medium blood vessels and ischemic and necrotic changes in subcutaneous tissue and in the skin. This disease occurs mostly in end-stage renal disease (ESRD) patients treated with dialysis (uremic calciphylaxis), however, there have been cases described in patients with preserved renal function (non-uremic calciphylaxis). Risk factors of vessel calcification are similar to those of atherosclerosis and include: obesity, hypertension, hypertriglyceridemia, increased concentration of LDL cholesterol and reduced concentration of HDL cholesterol. Calcification of vessels strongly correlates with prevalence of cardiovascular diseases and is an important predictive factor of cardiovascular events, including ischemic heart disease also with a fatal outcome [50]. In uremic rats, STS inhibited calcification in the heart and kidneys [51] and prevented nephrolithiasis [52]. Clinically, STS was used to treat calciphylaxis and was reported to reduce the mortality rate [53]. Many studies indicated that STS treatment caused quick pain relief in patients and accelerated healing of necrotic changes in the skin [54,55,56,57,58,59]. Chen at al. clearly demonstrated that STS directly inhibited high phosphorus-induced calcification in adipocytes but had no effect on vascular smooth muscle cell calcification [60]. Hayden and Goldsmith [61] have suggested that STS is a new hope for the treatment of calciphylaxis.

It should be reminded that the outer sulfur atom of thiosulfate exhibits properties characteristic of sulfane sulfur and that thiosulfate is a stable, nontoxic metabolite of H_2_S. In addition, currently, interrelations between the existing sulfur deposits in cells and endogenously produced H_2_S are increasingly often indicated. The synthesized H_2_S can be stored in the form of sulfane sulfur and can be released as needed by the cell. Sen et al. [62] demonstrated that STS can be considered for therapeutic intervention in heart failure due to its ability to modulate endogenous H_2_S generation. In addition, it was proven that H_2_S breathing in mice prevented inflammation and improved survival after lipopolysaccharide (LPS) challenge, which was associated with increased plasma thiosulfate level [63]. Sakaguchi et al. [64] showed that STS exerted robust anti-inflammatory effects in the mouse lung and vascular endothelium.

It appears that biological actions initially attributed to H_2_S actually are effects of sulfane sulfur compounds. Thus, it appears that rather thiosulfate which is a sulfane sulfur containing compound, than H_2_S, is a signaling agent possessing regulatory effect in biological systems [65]. Mishanina et al. [66] have suggested that thiosulfate is a signaling molecule, belonging to reactive sulfur species (RSS), which can be viewed as an ideal “Trojan horse”, because upon activation by sulfurtransferases, it can mobilize its outer sulfur to transfer.

Sulfane sulfur transfer to thiol groups of receptor and enzymatic proteins leads to the formation of persulfides and/or trisulfides that often changes activity of these proteins. This is another, besides *S*-thiolation and *S*-nitrosylation, mechanism of covalent modification of protein –SH groups [11]. The functions of sulfane sulfur include also posttranscriptional modification of transfer RNA (tRNA), and synthesis of iron-sulfur center in iron-sulfur proteins and coenzymes, such as biotin, lipoic acid and thiamine as well as involvement in antioxidant potential regulation [4].

## 6. Conclusions

**Figure 4:**
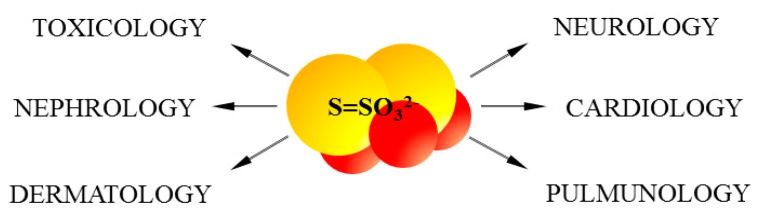
Thiosulfate and its therapeutic potential.

Aerobic and anaerobic cysteine transformations are linked via formation and biodegradation of thiosulfate. These processes occur in mitochondria and are regarded as a canonical H_2_S oxidation pathway (Figure 1). However, lately new chemical interactions between H_2_S and hemoproteins have been described, yielding also thiosulfate. This process was called a non-canonical H_2_S oxidation pathway (Figure 3). Until recently, it appeared that the goal of thiosulfate production was only to delay irreversible oxidation of H_2_S to sulfate excreted in urine. However, in the light of the newest data presented in this review, it seems that thiosulfate is a molecule that plays a much more prominent role in the human body (Figure 4).

The data reviewed in this paper also change the traditional understanding of Hb and Mb function as proteins engaged in binding, transport and storage of most of all oxygen molecule, and clearly show that these hemoproteins are important players in the H_2_S homeostatic processes significantly contributing to thiosulfate production. It is particularly important in RBCs which are devoid of the canonical mitochondrial H_2_S oxidation pathway, and thus in the whole circulatory system, and in exercising muscles, which, though possess mitochondria, produce energy in anaerobic processes running in the cytoplasm. It also appears that the non-canonical H_2_S oxidation pathway involving Ngb is the main H_2_S oxidation route in the brain.

We hope that all these findings will inspire further studies on the role of hemoproteins in the processes implicated in the formation of this fascinating molecule, which undoubtedly thiosulfate is, and on the mechanisms responsible for its biological activity in the human body.
